# Prevalence and Characteristic of Swine-Origin *mcr-1*-Positive *Escherichia coli* in Northeastern China

**DOI:** 10.3389/fmicb.2021.712707

**Published:** 2021-07-20

**Authors:** Ping Cheng, Yuqi Yang, Sai Cao, Haibin Liu, Xiaoting Li, Jichao Sun, Fulei Li, Muhammad Ishfaq, Xiuying Zhang

**Affiliations:** ^1^Heilongjiang Key Laboratory for Animal Disease Control and Pharmaceutical Development, Faculty of Basic Veterinary Science, College of Veterinary Medicine, Northeast Agricultural University, Harbin, China; ^2^Pharmacology Teaching and Research Department, School of Basic Medicine, Guizhou University of Traditional Chinese Medicine, Guiyang, China

**Keywords:** colistin resistance, *mcr-1* gene, swine, *Escherichia coli*, prevalence, characteristics

## Abstract

The emergence of the plasmid-mediated colistin resistance gene *mcr-1* is threatening the last-line role of colistin in human medicine. With *mcr-1*-positive *Escherichia coli* (*E. coli*) isolated from food animal being frequently reported in China, the prevalence of *mcr-1* in food animal has attracted public attention. In the present study, a total of 105 colistin-resistant *E. coli* strains were isolated from 200 fecal samples collected from six swine farms in northeastern China. *mcr*-PCR revealed that the prevalence of *mcr-1* in colistin-resistant *E. coli* was 53.33% (56/105). *mcr-1*-positive *E. coli* showed extensive antimicrobial resistance profiles with the presence of additional resistance genes, increased expression of multidrug efflux pump-associated genes, and increased biofilm formation ability. MLST differentiated all the *mcr-1-*positive *E. coli* into 25 sequence types (STs) and five unknown ST, and the most common ST was ST10 (*n* = 11). By phylogenetic group classification, the distribution of all *mcr-1*-positive *E. coli* belonging to groups A, B1, B2, and D was 46.43, 35.71, 5.36, and 5.36%, respectively. Conjugation experiment demonstrated that most of the *mcr-1* were transferable at frequencies of 2.68 × 10^–6^–3.73 × 10^–3^ among 30 representative *mcr*-*1-*positive *E. coli*. The plasmid replicon types IncI2 (*n* = 9), IncX4 (*n* = 5), IncHI2 (*n* = 3), IncN (*n* = 3), and IncP (*n* = 1) were detected in the transconjugants. The results of growth assay, competition experiment, and plasmid stability testing showed that acquisition of *mcr-1-*harboring plasmids could reduce the fitness of bacterial hosts, but *mcr-1* remained stable in the recipient strain. Due to the potential possibility of these *mcr-1*-positive *E. coli* being transmitted to humans through the food chain or through horizontal transmission, therefore, it is necessary to continuously monitor the prevalence and dissemination of *mcr-1* in food animal, particularly in swine.

## Introduction

The discovery and use of antibiotics in human medicine was regarded as one of the vast medical advancements over the past decades, and antibiotics also play an important role in food-animal agriculture (Worthington and Melander, 2013). The increasing amount of animal protein for human consumption accelerates the development of modern animal production. However, the widespread use of antibiotics in livestock has posed a significant public health threat, which can potentially increase selection pressure on antibiotic-resistant bacteria (ARB) and further promote the dissemination of ARB in livestock (You and Silbergeld, 2014). Moreover, the animal-origin ARB can be transmitted to humans through the environment and food chain as well as through direct contact (Graham et al., 2009).

*Escherichia coli* is one of the major pathogens in the swine industry, which is associated with gastrointestinal diseases and systemic infections, including diarrhea, edema disease, septicemia, polyserositis, mastitis, and urinary tract infections (Fairbrother et al., 2005). These diseases can lead to morbidity, mortality, and delayed growth, which are responsible for considerable economic losses and restrict the development of the swine industry. To maintain health and productivity, antibiotics are widely administered to treat *E. coli* infections in farms to swine *via* oral, either in feed or in water (Fairbrother et al., 2005). Among a variety of antibiotics used in swine farms, polypeptides and aminoglycosides are most frequently administrated (Sabine et al., 2017).

Colistin is a kind of cationic polypeptides and a member of the polymyxin family, including polymyxins A, B, C, D, and E. Only polymyxin B and polymyxin E (colistin) are currently used clinically. Due to the broad-spectrum activity against a wide range of Gram-negative bacteria (GNB), colistin is widely used in pig production to control intestinal infections caused by *Enterobacteriaceae* (Landman et al., 2008). The routine use of colistin in human medicine was abandoned in the 1970s due to its major side effects, including nephrotoxicity and neurotoxicity (Landman et al., 2008). However, with the emergence of multidrug-resistant Gram-negative bacteria (MDR-GNB) and the paucity of novel classes of antibiotics entering the clinic, colistin has been reintroduced to human clinical use as a last-line treatment option for severe infections caused by MDR-GNB (Falagas and Kasiakou, 2005). The rapid rise and dissemination of MDR-GNB led to the increased amounts of colistin used in humans and animals with the inevitable risk of accelerating the emergence of colistin resistance (Kempf et al., 2013).

Colistin resistance was commonly thought to be chromosomally mediated, until a novel plasmid-mediated colistin resistance gene *mcr-1* was characterized in *E. coli* isolated from animals and humans in China at the end of 2015 (Liu et al., 2016). Because of the rapid horizontal spread of colistin resistance by plasmids, the discovery of *mcr-1* has attracted public attention among physicians and veterinarians. To date, the cases of bacteria harboring *mcr-1* gene have been found in 47 different countries across six continents (Asia, Europe, Africa, North America, South America, and Oceania) from humans, animals, and environmental samples (Shi et al., 2020). Due to the high prevalence of *mcr-1*-positive *E. coli* originating from food animal than from humans, food animal production, particularly pig production, has been singled out as the major cause of *mcr-1* amplification and spread (Rhouma et al., 2016).

In this study, we aimed to investigate the prevalence and characteristics of *mcr-1* in swine farms in northeastern China by determining (1) the carriage rate of *mcr-1* in colistin-resistant *E. coli* isolated from swine fecal samples; (2) the antimicrobial resistance profiles of *mcr-1*-positive *E. coli* isolates; (3) the presence of additional resistance genes, the relative expression levels of multidrug efflux pump-associated genes, and biofilm formation ability in *mcr-1*-positive *E. coli* isolates; (4) the genetic relationship of the *mcr-1*-positive *E. coli* isolates by multilocus sequence typing (MLST) and phylogenetic group; and (5) the transferability, conjugation frequency, fitness cost, and plasmid stability of *mcr-1.*

## Materials and Methods

### Sample Collection and Bacterial Strain Identification

Between July 2016 and June 2017, a total of 200 fecal swabs were collected from six swine farms in northeastern China, including Heilongjiang (Harbin), Jilin (Changchun), and Liaoning (Shenyang). In each province, two geographically distinct swine farms were selected; 40 fecal swabs were randomly collected from 40 different pigs in each farm in Harbin, and 30 fecal swabs were randomly collected from 30 different pigs in each farm in Changchun and Shenyang. Fecal swabs were collected by placing a wet cotton swab at the animal anus of 2∼5 cm with minor rotation. The samples brought to the laboratory were immediately streaked out on MacConkey agar and incubated at 37°C for 18 h. The putative *E. coli* isolates on MacConkey agar (bright pink with a dimple) per sample were transferred to eosin methylene blue agar for further purification and were incubated at 37°C for 18 h. Randomly selected colonies with typical *E. coli* morphology were selected from each sample for PCR detection of *16S rRNA* gene and for sequencing (Seurinck et al., 2003). All confirmed *E. coli* isolates were stored at –80°C for further studies.

### Colistin Resistance Screening and Confirmation of *mcr-1*-Positive Strains

To isolate colistin-resistant *E. coli*, all the strains were screened on the MacConkey agar containing 2 μg/ml of colistin. The DNA templates of all colistin-resistant isolates were extracted using the DNA extraction kit (TIANGEN, Beijing, China) following the instructions of the manufacturer. The presence of *mcr-1* in colistin-resistant *E. coli* was determined by PCR amplification and followed by Sanger sequencing as described previously (Liu et al., 2016).

### Antimicrobial Susceptibility Testing

The susceptibility of all *mcr-1-*positive strains to 26 antibiotics, namely meropenem, ertapenem, imipenem, ampicillin, ampicillin–sulbactam, amoxicillin/clavulanic acid, cefuroxime, ceftazidime, cefepime, ceftriaxone, cefoxitin, aztreonam, gentamicin, amikacin, kanamycin, streptomycin, ciprofloxacin, levofloxacin, tetracycline, doxycycline, tigecycline, chloramphenicol, florfenicol, fosfomycin, sulfisoxazole, and nitrofurantoin, was determined by the standard disk diffusion method in accordance with the Clinical and Laboratory Standards Institute (CLSI); the interpretation of the susceptibility result was according to CLSI (document M100), 2018, except those for florfenicol and sulfisoxazole which were interpreted according to the CLSI VET01-A4, and tigecycline was interpreted in accordance with the European Committee on Antimicrobial Susceptibility Testing (EUCAST) 2017. The *E. coli* ATCC 25922 was used as a quality control strain.

### Detection of Additional Antimicrobial Resistance Genes

The presence of carbapenemase genes (*bla*_*KPC*_, *bla*_*NDM*_, *bla*_*OXA*__–__48_, and *bla*_*IMP*_) (Doyle et al., 2012), extended spectrum-β-lactamase (ESBL) genes (*bla*_*CTX*__–__*M*_) and non-ESBL genes (*bla*_*TEM*_, *bla*_*SHV*_, *bla*_*OXA*__–__1_) (Dallenne et al., 2010), pAmpC genes (*bla*_*CMY*_, *bla*_*FOX*_, and *bla*_*DHA*_) (Dallenne et al., 2010), tetracycline resistance genes [*tet*(A), *tet*(B), *tet*(C), and *tet*(M)] (Ng et al., 2001), aminoglycoside resistance genes [*rmtA*, *rmtB*, *rmtC*, *rmtD*, *armA*, *nmpA*, and *aac(3)-IV*] (Yeganeh Sefidan et al., 2019), fluoroquinolone resistance genes [*qnrA*, *qnrB*, *qnrC*, *qnrD*, *qnrS*, *oqxAB*, *qepA*, and *aac(6′)-Ib-cr*] (Ciesielczuk et al., 2013), streptomycin/spectinomycin resistance genes (*strA*, *strB*, and *aadA*) (Srinivasan et al., 2007), fosfomycin resistance genes (*fosA* and *fosA3*) (Lee et al., 2012), florfenicol resistance gene (*floR*) (Li et al., 2015), and sulfonamide resistance genes (*sul1*, *sul2*, and *sul3*) (Hammerum et al., 2006) was examined by PCR. The positive products were validated with Sanger sequencing, then all the obtained sequences were compared using Blast with those published in the NCBI database^1^.

### Phylogenetic Groups and Multilocus Sequence Typing Analysis

The genetic relatedness of *mcr-1-*positive strains was investigated by MLST as previously described for *E. coli* (Tartof et al., 2005). Furthermore, a two-step multiplex PCR was performed to determine the phylogenetic group, and the primers used (*chuA*, *yiaA*, and *TspE4.C2*) and details were the same as previously described (Clermont et al., 2000). Phylogenetic trees for all sequence types (STs) were constructed using the neighbor-joining method with MEGA software (Kumar et al., 2018). Annotation for each isolate and tree embellishment were visualized using Itol^2^.

### Detection of the Relative Expression Levels of Genes Encoding Efflux Pumps, Porins, and Regulators by Quantitative Real-Time PCR

Eleven representative strains were chosen from all *mcr*-*1-*positive *E. coli* for the detection of the relative expression levels of genes encoding efflux pumps (*acrA*, *mdfA*, *ydhE*, *acrE*, *tolC*, *mdtE*, and *mdtF*), regulators (*marA*, *soxS*, *fisF*, *dsrA*, and *evgA*), and porin protein-encoding genes (*ompC* and *ompF*). Total RNA of *mcr-1*-positive strains and a reference strain *E. coli* ATCC 25922 was extracted using TRIzol reagent (Thermo Fisher Scientific, Waltham, MA, United States), and cDNA was synthesized with 5 × All-In-One MasterMix (ABM, Richmond, Canada) following the instructions of the manufacturer. The *mdh* gene was used as the housekeeping gene. Quantitative real-time PCR (BioEasy SYBR Green High ROX Master Mix, Bioer, Hangzhou, China) was performed according to the methods described by Vinué et al. (2015). The relative expression levels of the tested genes were calculated using the 2^–ΔΔ*CT*^ method as described by Huang W. et al. (2020).

### Detection of Biofilm Formation Ability

All *mcr-1*-positive isolates were inoculated into 15 ml tubes containing 5 ml Luria–Bertani (LB) broth and then cultured overnight in a shaking incubator at 37°C. The biofilm formation assay of these isolates was then conducted in 96-well polystyrene flat-bottom microtiter plates as described previously (Teh et al., 2010). To quantify the biofilm formation ability, the absorbance values of the solution were measured at 590 nm using an automated Multiskan FC reader (Thermo Fisher Scientific). The experiment was repeated independently three times.

### Conjugation Experiment and Plasmid Replicon-Type Analysis

The transferability of *mcr-1* was tested by conjugation experiment with *mcr-1-*positive *E. coli* as donors and rifampicin-resistant *E. coli* EC600 as recipient strains. The MacConkey agar plates containing rifampicin (256 μg/ml) and colistin (2 μg/ml) were used to select *mcr*-*1-*positive transconjugants. PCR analysis and DNA sequencing were carried out to confirm that transconjugants were derivatives of the recipient strain *E. coli* EC600. The transfer frequency of *mcr-1* was determined as described in a previous study (Liu et al., 2016). The replicon types of the transconjugants were determined according to previous studies (Carattoli et al., 2005; Johnson et al., 2012).

### Growth Assay and *in vitro* Competition Experiment

To assess the fitness impact of *mcr-1* carriage on the host, growth assay and *in vitro* competition experiment were carried out. Growth curves for the recipient (EC600) and *mcr-1*-positive *E. coli* transconjugants were performed in 96-well flat-bottom plates (Corning Inc., Corning, NY, United States) as described previously (Long et al., 2019). *In vitro* competition experiments were conducted using *mcr-1*-positive *E. coli* transconjugants competing with EC600. Twenty-four-hour competition experiments were performed as described previously (He et al., 2017). Growth assay and *in vitro* competition experiment were performed in triplicate.

### Plasmid Stability Testing

To estimate the stability of the plasmid harboring *mcr-1*, plasmid stability experiments were performed using *mcr-1*-positive *E. coli* transconjugants as described previously (Sota et al., 2010).

## Results

### Prevalence of *mcr-1* in Colistin-Resistant *E. coli*

A total of 176 *E. coli* strains were isolated from 200 fecal samples collected from six swine farms located in northeastern China, and the *E. coli* isolates showed high resistance rate to colistin (59.66%, 105/176). Colistin-resistant *E. coli* colonies were identified in 66.20% (47/71), 54.90% (28/51), and 55.56% (30/54) *E. coli* strains isolated from swine farms in Heilongjiang, Jilin, and Liaoning, respectively. *mcr*-PCR and sequencing revealed that 56 *E. coli* were positive for *mcr-1*, the carriage rate was extremely high (53.33%, 56/105), and the prevalence of *mcr-1* in colistin-resistant *E. coli* isolated from swine farms in Heilongjiang, Jilin, and Liaoning was 46.81% (22/47), 53.57% (15/28), and 63.33% (19/30), respectively.

### Antimicrobial Susceptibility of *mcr-1-*Positive *E. coli*

**FIGURE 1:**
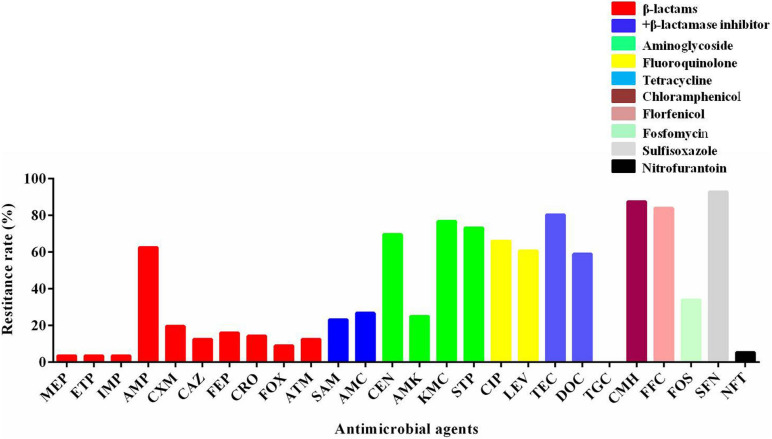
The resistant rate of *mcr-1-*positive *E. coli* to other antibiotics. MEP, meropenem; ETP, ertapenem; IMP, imipenem; AMP, ampicillin; CXM, cefuroxime; CAZ, ceftazidime; FEP, cefepime; CRO, ceftriaxone; FOX, cefoxitin; ATM, aztreonam; SAM, ampicillin–sulbactam; AMC, amoxicillin–clavulanic acid; GEN, gentamicin; AMK, amikacin; KMC, kanamycin; STP, streptomycin; CIP, ciprofloxacin; LEV, levofloxacin; TEC, tetracycline; DOC, doxycycline; TGC, tigecycline; CMH, chloramphenicol; FFC, florfenicol; FOS, fosfomycin; SFN, sulfisoxazole; NFT, nitrofurantoin.

The susceptibility of 56 *mcr-1-*positive *E. coli* isolates to other antimicrobials was determined. The percentages of resistance rate are presented in Figure 1. There were a high rate of resistance (60–100%) to gentamicin, kanamycin, streptomycin, ciprofloxacin, levofloxacin, tetracycline, chloramphenicol, florfenicol, doxycycline, and sulfisoxazole; a moderate rate of resistance (20–60%) to ampicillin, ampicillin–sulbactam, amoxicillin–clavulanic acid, amikacin, and fosfomycin; and a low rate of resistance (<20%) to meropenem, ertapenem, imipenem, cefuroxime, ceftazidime, cefepime, ceftriaxone, cefoxitin, aztreonam, and nitrofurantoin. There were no strains that were resistant to tigecycline. As shown in Table 1, most of the *mcr-1-*positive *E. coli* were multidrug resistant.

**TABLE 1:** Characteristics and antimicrobial resistance profiles of *mcr-1*-positive *E. coli.*

Strains	ST	Phylogroup	Antimicrobial resistance
HLJ173	1,421	B1	ATM/GEN/KMC/STP/CIP/LEV/TEC/CMH/SFN
LN58	410	A	AMP/CAZ/FEP/CRO/FOX/GEN/KMC/STP/CIP/LEV/TEC/DOC/CMH/FFC/SFN
LN122	1,463	B1	AMP/SAM/AMC/CIP/LEV/TEC/SFN
LN191	20	B1	GEN/KMC/STP/CIP/LEV/SFN
LN252	20	A	AMP/SAM/AMC/GEN/KMC/CIP/LEV/DOC/CMH/FFC/SFN/AMK
JL124	5,229	B1	AMP/GEN/KMC/STP/CIP/LEV/TEC/DOC/CMH/FFC/SFN/AMK
HLJ226	New ST1	B1	GEN/KMC/STP/CIP/LEV/CMH/FFC/SFN/FOS
LN72	1,0580	A	TEC/CMH/FFC/SFN/FOS
LN176	93	A	AMP/ATM/SAM/AMC/GEN/KMC/STP/CIP/LEV/TEC/DOC/CMH/FFC/SFN
JL226	New ST4	B1	AMP/SAM/AMC/GEN/KMC/STP/CIP/TEC/DOC/CMH/FFC/SFN/NFT/AMK
JL125	48	A	GEN/KMC/STP/DOC/CMH/FFC/SFN/FOS
HLJ8	10	Unknown	AMP/CXM/CAZ/FEP/CRO/GEN/KMC/CIP/TEC/DOC/CMH/FFC/SFN/AMK
JL252	9,159	Unknown	GEN/KMC/STP/CMH/FFC/SFN/FOS
LN74	10	A	AMC/SFN
HLJ212	10	A	AMP/TEC/DOC/CMH/FFC/SFN/FOS
HLJ84	New ST5	B1	AMP/GEN/KMC/STP/CIP/LEV/TEC/CMH/FFC/SFN/AMK
LN20	617	A	GEN/KMC/STP/TEC/DOC/CMH/FFC/SFN
LN203	2,935	B1	KMC/STP/CIP/SFN
JL114	3,944	A	AMP/CAZ/FEP/CRO/LEV/TEC/DOC/CMH/FFC/SFN/FOS
HLJ464	3,944	A	AMP/FEP/LEV/DOC/FOS
LN221	3,944	B1	SAM/FOS
LN220	398	B1	AMP/SAM/AMC/KMC/STP/CIP/LEV/DOC/CMH/FFC/SFN
JL7	3,014	B2	AMP/SAM/AMC/CMH/FFC/SFN/FOS
JL127	1,421	B1	SAM/AMC/GEN/KMC/STP/CIP/LEV/DOC/SFN/AMK
HLJ174	3,856	A	CIP/LEV/TEC/DOC/SFN
HLJ456	New ST3	B1	TEC/DOC/CMH/FFC/SFN
HLJ438	New ST2	B1	AMP/CAZ/FEP/CRO/FOX/GEN/KMC/STP/TEC/CMH/FFC/FOS
HLJ56	4,379	B1	GEN/KMC/STP/CIP/LEV/TEC/DOC/CMH/FFC/SFN
LN106	156	B2	AMP/CXM/SAM/AMCGEN/KMC/STP/CIP/LEV/TEC/DOC/CMH/FFC/SFN/AMK
LN19	1,589	A	GEN/KMC/STP/CIP/LEV/TEC/DOC/CMH/FFC/SFN
HLJ63	410	A	AMP/CXM/CAZ/FEP/CRO/FOX/TEC/DOC/CMH/FFC/SFN/FOS
LN251	20	B1	CIP/LEV/TEC/DOC/CMH/FFC/SFN
JL128	5,229	B1	ATM/AMC/GEN/KMC/STP/CIP/LEV/TEC/DOC/CMH/FFC/SFN
JL47	5,229	B1	AMP/GEN/KMC/STP/CIP/LEV/TEC/SFN/AMK/FOS
HLJ70	898	B1	CXM/ATM/KMC/STP/CIP/LEV/TEC/DOC/CMH/FFC/SFN
HLJ43	898	A	AMP/SAM/AMC/GEN/KMC/STP/CIP/LEV/TEC/CMH/FFC/SFN/FOS
JL43	224	D	CXM/GEN/KMC/STP/CIP/LEV/TEC/DOC/CMH/FFC/SFN/NFT
LN66	131	D	GEN/KMC/STP/TEC/CMH/FFC/SFN
LN186	93	B1	AMP/CXM/ATM/AMC/GEN/KMC/STP/CIP/LEV/TEC/CMH/FFC/SFN/AMK/FOS
JL55	48	A	AMP/SAM/GEN/KMC/STP/CIP/LEV/TEC/DOC/CMH/FFC/SFN
JL63	48	A	AMP/GEN/KMC/STP/CIP/LEV/TEC/DOC/CMH/FFC/SFN
HLJ79	48	A	AMP/GEN/KMC/STP/TEC/CMH/FFC/SFN/AMK/FOS
HLJ194	772	B2	AMP/ATM/GEN/KMC/STP/TEC/DOC/CMH/FFC/FOS
LN190	772	A	AMP/CXM/TEC/DOC/CMH/FFC/SFN
HLJ188	772	Unknown	CXM/GEN/KMC/STP/TEC/DOC/CMH/FFC/SFN/AMK
LN59	617	B1	AMP/CXM/CAZ/FEP/CRO/GEN/KMC/STP/CIP/LEV/TEC/CMH/FFC/SFN/AMK
JL176	165	A	AMP/GEN/KMC/STP/CIP/LEV/TEC/DOC/CMH/FFC
HLJ187	6,730	Unknown	AMP/GEN/KMC/STP/CIP/LEV/TEC/DOC/CMH/FFC/SFN
HLJ336	10	A	AMP/GEN/KMC/STP/CIP/LEV/TEC/DOC/CMH/FFC/SFN
JL9	10	A	AMP/GEN/KMC/STP/CIP/LEV/TEC/DOC/CMH/FFC/SFN
JL33	10	A	AMP/CXM/FEP/CRO/FOX/GEN/KMC/STP/CIP/LEV/TEC/DOC/CMH/FFC/NFT/AMK/FOS
HLJ230	10	D	MEP/ETP/IMP/AMP/CXM/CAZ/FEP/CRO/FOX/GEN/KMC/STP/TEC/CMH/FFC/FOS/SFN
HLJ222	10	A	AMP/SAM/GEN/KMC/STP/CIP/LEV/TEC/DOC/CMH/FFC/SFN/FOS
LN182	10	A	AMP/SAM/AMC/GEN/KMC/STP/CIP/LEV/TEC/CMH/FFC/SFN/FOS
LN215	10	A	AMP/ATM/GEN/KMC/STP/CIP/LEV/TEC/CMH/FFC/SFN
JL79	10	D	AMP/SAM/AMC/KMC/STP/TEC/CMH/FFC/FOS/SFN

### Presence of Additional Resistance Genes in *mcr-1-*Positive *E. coli*

**FIGURE 2:**
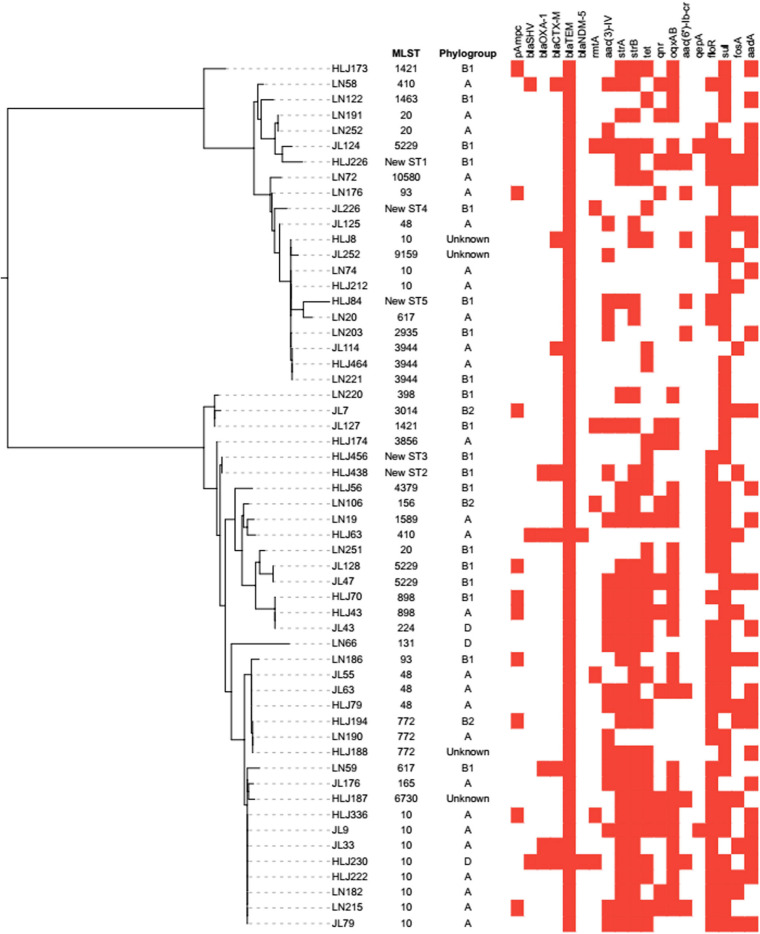
Analysis of phylogroup, antimicrobial resistance genes, and relationships among 56 *mcr-1*-positive *E. coli* isolates from swine farms in northeastern China. Relationships among 56 *mcr-1*-positive *E. coli* isolates are indicated using an unrooted tree based on the alignments of concatenated MLST allelic sequences using the neighbor-joining method. For comparison of resistance genes among the 56 *mcr-1*-positive *E. coli* isolates, the red squares represent positivity for antimicrobial resistance genes.

Molecular features revealed that most *mcr-1-*positive *E. coli* carried additional resistance genes, as shown in Figure 2. Overall, *bla*_*TEM*_ (*n* = 56, 100%) was the most common non-ESBL gene in our study, followed by *bla*_*SHV*__–__1_ and *bla*_*OXA*__–__1_ that were identified in three (5.36%) and five (8.93%) isolates, respectively. In addition, the ESBL gene *bla*_*CTX*__–__*M*_ was detected in eight (14.86%) *mcr-1*-positive *E. coli* isolates. The detected pAmpC genes were *bla*_*CMY*_ (*n* = 10, 17.86%), *bla*_*FOX*__–__5_ (*n* = 5, 8.93%), and *bla*_*DHA*__–__1_ (*n* = 2, 3.57%). The carbapenemase genes (*bla*_*KPC*_, *bla*_*OXA*_, and *bla*_*IMP*_) were not detected, and only *bla*_*NDM*__–__5_ was detected in two (3.57%) isolates. Among aminoglycoside resistance genes, only *rmtA* [7, 12.50%) and *aac(3)-IV* (25, 44.64%] were detected. As for fluoroquinolone resistance genes, there were 10 (17.86%), 7 (12.50%), 31 (55.36%), 2 (3.57%), and 9 (16.07%) isolates harboring *qnrD*, *qnrS*, *oqxAB*, *qepA*, and *aac(6′)-Ib-cr*, respectively, but there were no strains harboring *qnrA*, *qnrB*, and *qnr*C. The number of isolates harboring *tet*(A), *tet*(B), and *tet*(M) was 27 (48.21%), 20 (35.71%), and 31 (55.36%), respectively. The plasmid-encoded *floR* gene that conferred chloramphenicol resistance was detected in 38 (67.86%) *mcr-1-*positive strains. The isolates positive for sulfonamide resistance genes comprised 33 (58.93%) strains harboring *sul1*, followed by 24 (42.86%) and 18 (32.14%) strains harboring *sul2* and *sul3*, respectively. The *strA* and *strB* were closely associated with streptomycin resistance, which were detected in 34 (60.71%) and 39 (69.64%) isolates. The *fosA* (*n* = 9, 16.07%) and *fosA3* (*n* = 17, 30.36%) were prevalent in fosfomycin-resistant isolates.

### Molecular Genotyping of *mcr-1-*Positive *E. coli*

**FIGURE 3:**
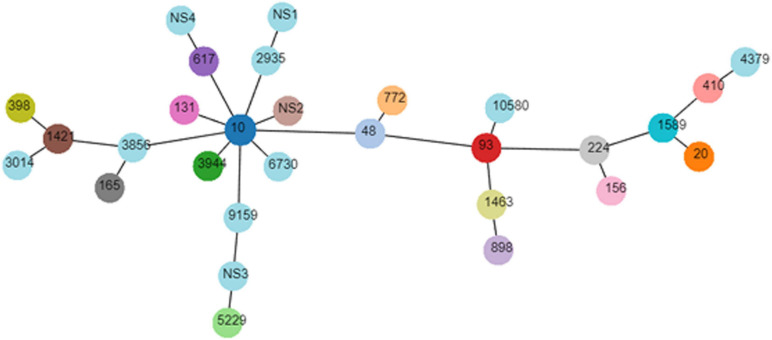
Minimal spanning tree of *mcr-1-*positive *E. coli.* Each circle corresponds to one ST and the size of each circle indicated the number of isolates in this ST type.

The genotyping results of *mcr-1*-positive *E. coli* are summarized in Table 2. The *mcr-1-*positive isolates were distributed into phylogroups A (*n* = 26), B1 (*n* = 20), B2 (*n* = 3), and D (*n* = 3), and the phylogroup was undefined for four isolates. MLST differentiated the 56 *mcr-1-*positive *E. coli* into 25 STs and five unknown ST (untypable). As shown in Figure 3, the most common ST was ST10 (*n* = 11), followed by ST48 (*n* = 4), ST20 (*n* = 3), ST3944 (*n* = 3), ST772 (*n* = 3), ST5229 (*n* = 3), ST617 (*n* = 2), ST410 (*n* = 2), ST93 (*n* = 2), ST898 (*n* = 2), and ST1421 (*n* = 2), and then by single ST isolates, including ST165, ST10580, ST3856, ST1589, ST398, ST1463, ST4379, ST2935, ST156, ST3014, ST131, ST224, ST6730, and ST9159. Moreover, ST10, ST48, and ST617 are different by one or two alleles and they correspond to clonal complex CC10. As shown in Figure 2, phylogenetic analysis of all *mcr-1-*positive *E. coli* underlined the evidence for the horizontal transfer of *mcr-1.*

**TABLE 2:** Genotyping of *mcr-1-*positive *E. coli.*

Phylogroup (number of strains)	Clonal complex (number of strains)	Sequence type (number of strains)
A (26)	CC10 (14)	ST10 (9), ST48 (4), ST617 (1)
	CC20 (1)	ST20 (1)
	CC23 (2)	ST410 (2)
	CC165 (1)	ST165 (1)
	CC168 (1)	ST93 (1)
	Other CC (7)	ST3944 (2), ST10580 (1), ST3856 (1), ST898 (1), ST772 (1), ST1589 (1)
B1 (20)	CC10 (1)	ST617 (1)
	CC20 (2)	ST20 (2)
	CC101 (3)	ST5229 (3)
	CC168 (1)	ST93 (1)
	CC398 (1)	ST398 (1)
	Other CC (12)	NS1 (1), ST898 (1), NS2 (1), ST1421 (2), NS3 (1), ST3944 (1), ST1463 (1), ST4379 (1), NS4 (1), ST2935 (1), NS5 (1)
B2 (3)	CC156 (1)	ST156 (1)
	Other CC (2)	ST772 (1), ST3014 (1)
D (3)	CC10 (1)	ST10 (1)
	CC131 (1)	ST131 (1)
	Other CC (1)	ST224 (1)
Unknown (4)	CC10 (1)	ST10 (1)
	Other CC (3)	ST6730 (1), ST9159 (1), ST772 (1)

### Relative Expression Levels of Genes Encoding Efflux Pumps, Porins, and Regulators in *mcr-1*-Positive *E. coli*

**FIGURE 4:**
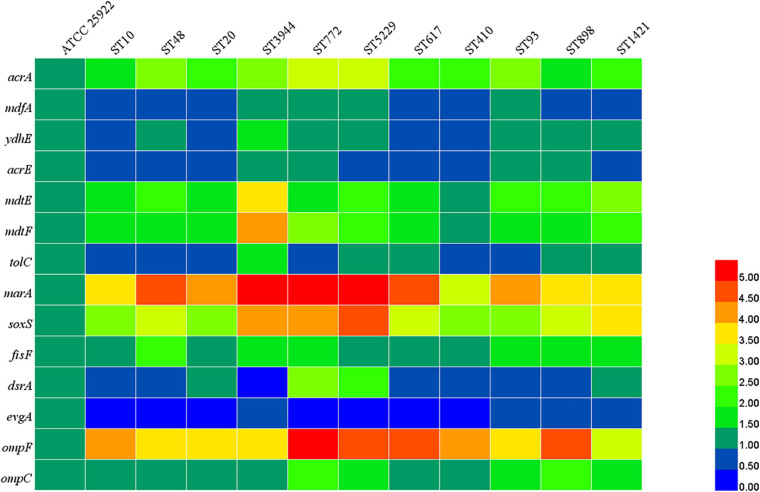
Relative expression levels of efflux pumps, porins, and regulators. The different colors indicate the different expression levels.

According to the results of MLST, 11 STs were predominant among *mcr-1*-positive *E. coli*. One representative strain of *E. coli* was chosen from each ST for subsequent detection. As shown in Figure 4, compared with the *E. coli* ATCC 25922, the relative expression levels of *acrA*, *mdtE*, *mdtF*, *marA*, *soxS*, *fisF*, *ompF*, and *ompC* were increased in all tested *mcr*-*1-*positive *E. coli*. The expression of *mdfA*, *ydhE*, *acrE*, *tolC*, and *dsrA* was increased in four, seven, four, five, and four strains, respectively, and the expression of *evgA* was reduced in all tested *mcr*-*1-*positive *E. coli*. The results indicated that upregulation of the expression of efflux pump-related genes could be used to explain the multidrug resistance of *mcr-1*-positive *E. coli*.

### Biofilm Formation Ability of *mcr-1*-Positive *E. coli*

**FIGURE 5:**
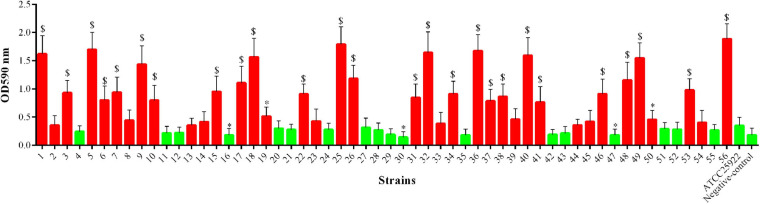
Biofilm formation ability of *mcr-1*-positive *E. coli*; the red indicates increased biofilm formation ability, and green indicates decreased biofilm formation ability compared with *E. coli* ATCC 25922. ^∗^*p* < 0.05; ^$^*p* < 0.01.

As shown in Figure 5, among the 56 *mcr-1*-positive *E. coli* strains, 28 (50.00%) strains showed significantly increased ability of biofilm formation compared with the *E. coli* ATCC 25922 (*p* < 0.05 or *p* < 0.01), and two (3.57%) strains showed significantly decreased ability of biofilm formation (*p* < 0.05). However, the remaining strains (26/56, 46.43%) showed no significant changes in their ability of biofilm formation.

### Transferability of *mcr-1* and Plasmid Replicon Types in *mcr-1-*Positive Transconjugants

The transferability of *mcr-1* and conjugation frequencies are exhibited in Table 3. Among 30 representative *mcr-1-*positive *E. coli*, majority of the strains (*n* = 26) were capable of transferring *mcr-1* to the recipient rifampicin-resistant *E. coli* EC600. The conjugation frequencies of the isolates lay between 2.68 × 10^–6^ and 3.73 × 10^–3^. The detected plasmid replicon types in the transconjugants included IncI2 (*n* = 8), IncX4 (*n* = 5), IncHI2 (*n* = 3), IncN (*n* = 3), and IncP (*n* = 1).

**TABLE 3:** MLST, transferability, conjugation efficiencies, and plasmid replicon types of 30 *mcr-1-*positive *E. coli.*

Strains	Phylogroup	*mcr-1*	Sequence type	Transferability	Conjugation efficiency	Plasmid replicon types	MIC* (μg/ml)
HLJ8	Unknown	+	10	+	1.85 × 10^–4^	IncHI2	8
HLJ63	A	+	410	+	3.73 × 10^–3^	IncP/IncHI2	4
HLJ70	B1	+	898	+	1.62 × 10^–4^	IncN	4
HLJ173	B1	+	1,421	+	1.97 × 10^–4^	IncN/IncX4	2
HLJ194	B2	+	772	–	−	−	−
HLJ187	Unknown	+	6,730	+	2.85 × 10^–3^	IncX4	8
HLJ79	A	+	48	+	3.15 × 10^–4^	IncI2	8
HLJ56	B1	+	4,379	+	5.36 × 10^–4^	IncHI2	4
HLJ174	A	+	3,856	+	2.64 × 10^–4^	IncI2	4
HLJ464	A	+	3,944	+	3.18 × 10^–4^	IncX4	4
JL124	B1	+	5,229	–	−	−	−
HLJ226	B1	+	NewST 1	+	5.14 × 10^–6^	IncN/IncX4	2
JL176	A	+	165	+	3.24 × 10^–4^	IncN	4
JL252	Unknown	+	9,159	+	2.76 × 10^–4^	IncI2	4
JL7	B2	+	3,014	+	1.96 × 10^–3^	IncHI2	4
JL43	D	+	224	–	−	−	−
HLJ438	B1	+	NewST 2	+	3.62 × 10^–4^	IncX4	2
HLJ456	B1	+	NewST 3	+	2.84 × 10^–4^	IncI2	2
JL226	B1	+	NewST 4	+	2.26 × 10^–5^	IncP	4
HLJ84	B1	+	NewST 5	+	4.81 × 10^–4^	IncI2	4
LN20	A	+	617	+	3.67 × 10^–4^	IncP/IncHI2	4
LN186	B1	+	93	+	2.53 × 10^–4^	IncI2	8
LN203	B1	+	2,935	+	2.68 × 10^–6^	IncN	2
LN66	D	+	131	–	−	−	−
LN72	A	+	10,580	+	5.64 × 10^–5^	IncI2	8
LN106	B2	+	156	+	2.37 × 10^–4^	IncX4	4
LN122	B1	+	1,463	+	2.98 × 10^–4^	IncI2	4
LN252	A	+	20	+	2.75 × 10^–4^	IncI2/IncX4/IncHI2	4
LN220	B1	+	398	+	2.12 × 10^–4^	IncP	4
LN19	A	+	1,589	+	9.05 × 10^–5^	IncX4	4

*The MIC of colistin against mcr-1-positive transconjugants.

The combinations of IncN/IncX4 (*n* = 2), IncP/IncHI2 (*n* = 2), and IncI2/IncX4/IncHI2 (*n* = 1) were detected, indicating some transconjugants harbored several replicon types.

### Fitness Cost and Plasmid Stability

**FIGURE 6:**
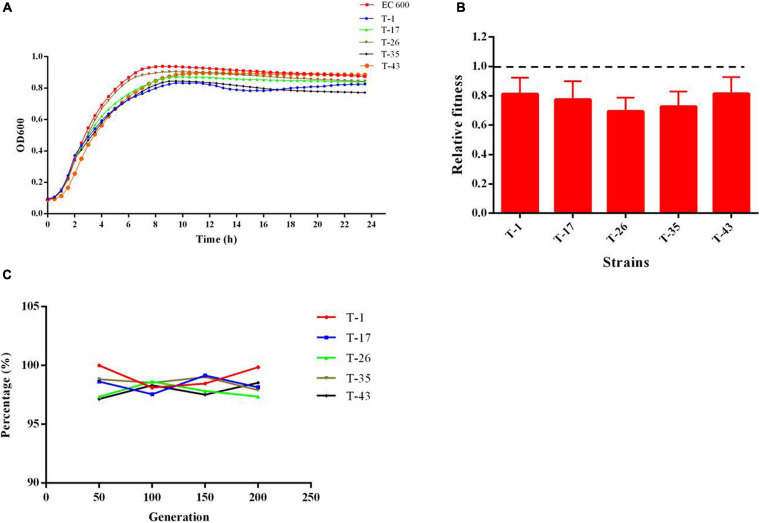
**(A)** Growth kinetics of transconjugants harboring *mcr-1*; **(B)** relative fitness of transconjugants harboring *mcr-1*, a relative fitness of 1 indicates that the transcoujugants undergo no fitness cost; **(C)** stability of plasmid harboring *mcr-1* in transconjugants.

As shown in the growth curves of Figure 6A, compared with the recipient (EC600), the growth rates at growth phase and cell densities at stationary phase were decreased slightly in *mcr-1*-positive *E. coli* transconjugants. The results of the *in vitro* competition experiment (Figure 6B) showed that the relative fitness values of all selected *mcr-1*-positive *E. coli* transconjugants were below 1. These results revealed that the acquisition of *mcr-1*-bearing plasmid could place an energy burden on the bacterial host and incur fitness cost. A total of five *mcr-1*-positive *E. coli* transconjugants were randomly selected and were passaged daily for 10 days in the absence of antibiotic selection. The results (Figure 6C) showed that *mcr-1* could be detected in transconjugants after a series of passages, suggesting that the plasmid harboring *mcr-1* remains stable in the hosts.

## Discussion

In the 1960s, several countries permitted the use of colistin in food animal production (Rhouma et al., 2016). However, the regular use of colistin in food animal is recognized as one of the major contributors to the emergence of colistin-resistant *Enterobacteriaceae* in humans (Maamar et al., 2018). The discovery of a novel stable plasmid-mediated gene *mcr-1* in *E. coli* contributed to our understanding of potential colistin resistance transmission between animals and humans (Liu et al., 2016). Moreover, livestock and poultry have been described as the major reservoir for colistin resistance (Rhouma et al., 2016). A survey has been performed to investigate the prevalence of colistin resistance in *E. coli* isolated from farms in different geographic areas of China during 2013–2014, which revealed that colistin resistance rates in *E. coli* from pigs, chickens, and cattle were 26.5, 14.0, and 0.9%, respectively (Zhang et al., 2019). The results demonstrated that colistin resistance was extremely serious in food animals, particularly in pigs.

In this study, *E. coli* strains isolated from swine farms in northeastern China showed significantly higher frequency of colistin resistance (52.5%). This result supports a previous finding that colistin resistance in *E. coli* occurred widely in pigs (54.25%) in intensive breeding farms of Jiangsu Province from 2015 to 2016 (Zhang et al., 2019). The high frequency of colistin resistance in the *E. coli* isolates recovered from food production animals could be explained by the increasing amount of colistin administrated in animal husbandry in the past few years, especially in swine (Zhang et al., 2019). It has been reported that colistin was used in massive quantities in the swine industry for the treatment of gastrointestinal disease worldwide, including France, Belgium, Spain, Austria, Germany, and China (Rhouma et al., 2016). Moreover, the amount of colistin used in agriculture was 11,942 tons per year by the end of 2015 in China, which was predominant all over the world (Liu et al., 2016).

The rapid horizontal spread of *mcr-1* by plasmids is one of the major reasons for the increasing prevalence of colistin resistance. Several studies have reported that many countries and regions found the presence of GNB carrying *mcr-1* in humans, animals, and the environment (Fernandes et al., 2016; Hadjadj et al., 2017). In this study, 56 (53.33%) *E. coli* strains were positive for *mcr-1* among 105 colistin-resistant *E. coli* isolated from swine farms. Similar to our result, a surveillance of colistin resistance performed in Jiangsu Province revealed that the *mcr-1* prevalence was 68.86% in pigs (Zhang et al., 2019). A previous study showed a high *mcr-1*-positive rate (79.2%) in swine-origin *E. coli* isolated from nine provinces in China. Further testing showed that most *mcr-1*-positive bacteria were identified as *E. coli*, demonstrating that *E. coli* was the predominant bacterial host of the *mcr-1* gene (Zhang et al., 2018). With the purpose of promoting growth, colistin had been widely used as a feed additive in farms for many years in China before 2017. The excessive use of colistin potentially increases the selection pressure which can promote the spread of *mcr-1*, finally leading to an exceedingly high prevalence of *mcr-1* in food animals (Tong et al., 2018). Fortunately, the Chinese government has banned the use of colistin as food additive for growth promotion in farms since April 1, 2017.

It has been reported that plasmids harboring *mcr-1* usually carry other resistance genes, encoded for aminoglycosides, quinolones, etc. (Rozwandowicz et al., 2018). Furthermore, the resistance genes can be horizontally transferred *via* plasmids, which is recognized as one of the major reasons for the extensive resistance profiles of the *mcr-1*-positive bacteria (Fan et al., 2020). In the present study, *mcr-1-*positive *E. coli* isolates displayed high resistance rates to antibiotics that are commonly used in veterinary medicine, including florfenicol, doxycycline, ciprofloxacin, chloramphenicol, streptomycin, gentamicin, kanamycin, and ampicillin. They showed low rates of resistance to some important antibiotics in human medicine, such as tigecycline, nitrofurantoin, ertapenem, meropenem, and imipenem. The usage of different antibiotics may lead to various resistance profiles, and antibiotics commonly used in food animals can form selection pressure on bacteria to become resistant. The antimicrobial resistance profiles of *mcr*-*1-*positive *E. coli* in this study were similar to the large-scale investigation performed in China (Huang et al., 2017).

The emergence of a superbug resistant to all last-line antibiotics (carbopenems, colistin, and tigecycline) was rare in swine farms, and a similar result was also obtained in a previous study about *E. coli* of food-animal origin in China (Tong et al., 2018). However, co-carriage of *mcr-1* and *bla*_*NDM*__–__5_ was detected in this study which has been found in *Enterobacteriaceae* isolated from animals and humans (Du et al., 2016; Paveenkittiporn et al., 2020). Notably, the one isolate harboring *mcr-1* and *bla*_*NDM*__–__5_ belongs to phylogroup D, indicating the possibility of two isolates being pathogenic *E. coli* responsible for extraintestinal infection (Khanawapee et al., 2020). The extensive resistance profiles of *mcr*-*1-*positive *E. coli* could be explained by the high frequencies of the presence of other resistance genes, including *bla*_*TEM*_, *bla*_*CTX*__–__*M*_, *aac3-IV*, *tet*(A), *tet*(M), *floR*, *sul1*, *sul2*, and *oqxAB*. Multidrug efflux pump in bacteria is a ubiquitous mechanism leading to cross-resistance with several antimicrobial agents and can increase the resistance level by interacting synergistically with other resistance mechanisms (Baron and Rolain, 2018). It has been demonstrated that β-lactams, fluoroquinolones, tetracycline, and chloramphenicol could be the substrates of efflux pumps. In the present study, the relative expression levels of some genes associated with multidrug efflux pumps were increased in *mcr-1*-positive *E. coli*. When the same plasmid carries *mcr-1* and various resistance genes, the frequent use of other antibiotics, such as aminoglycosides, tetracyclines, or sulfonamides, also can promote the selection of colistin resistance (Sabine et al., 2017). Therefore, we cannot ignore the effect of the high prevalence of *mcr-1* in swine-origin *E. coli*, increasing the number of multidrug-resistant bacteria.

Biofilm formation is commonly relied on regarding the cooperation of different bacterial strains and species for a common goal. Biofilm shows as bacteria form dense surface-associated communities, which could allow them to prosper and protect each other; bacteria within a biofilm showed enhanced tolerance to harsh environmental conditions and increased antibiotic resistance (Rabin et al., 2015). It has been suspected that biofilm could play a significant role in the persistence of bacterial infections in both clinical and food industries (Bridier et al., 2015). Unfortunately, most of the *mcr-1*-positive *E. coli* isolated from swine in this study were biofilm producers. The result suggested that biofilm formation is one of the strategies used by these bacteria against antibiotics and environmental stress. The prevalence of biofilm in swine-origin *mcr-1*-positive *E. coli* maybe associated with the excessive use of antibiotics in swine farms. A similar idea has been reported that the improper use of antibiotics may select for and further accumulate bacteria with a strong or moderate biofilm formation ability (Ma et al., 2020).

Many studies have demonstrated that the mobile genetic element of *mcr-1* could promote colistin resistance dissemination between animals and humans and result in the high prevalence of *mcr-1* worldwide (Liu et al., 2016; Wang et al., 2018). In this study, the transferability and the dissemination risk of *mcr-1* were assessed among 30 representative strains. The results were in line with previous findings which showed that majority of the reported *mcr* encoded by plasmids were transferable (Wang et al., 2018). Among the reported *mcr-1*, majority of them were mediated by plasmids, but there were some studies that reported the emergence of *mcr-1* on chromosome, or the plasmids harboring *mcr-1* were inconjugative, which could lead to failure of horizontal transfer (Lu et al., 2019).

The plasmid replicon types IncI2 (*n* = 9), IncX4 (*n* = 5), IncHI2 (*n* = 3), IncN (*n* = 3), and IncP (*n* = 1) were detected in the transconjugants. Among the already reported plasmids harboring the *mcr-1* gene, they belong to different replicon types, including IncI2, IncHI1, IncHI2, IncFIB, IncFII, IncP, IncX4, and IncY (Huang H. et al., 2020). With the use of colistin in clinical settings, the type of plasmids carrying *mcr-1* became more diverse which was reported by a survey performed in China to investigate the carriage of *mcr-1* among hospital patients, suggesting that colistin administration could promote the dissemination of diverse resistance plasmids among *E. coli* isolates (Huang H. et al., 2020). Moreover, the combinations of IncN/IncX4 (*n* = 2), IncP/IncHI2 (*n* = 2), and IncI2/IncX4/IncHI2 (*n* = 1) were detected, indicating that some transconjugants harbored several replicon types. This could be explained by the co-transfer of *mcr-1* and other resistance genes. The results of growth assay analysis and *in vitro* competition experiment showed that the acquisition of *mcr-1-*harboring plasmids could reduce the fitness of the bacterial host, but plasmid stability testing revealed that *mcr-1-*harboring plasmids remained stable in the recipient strain, which was consistent with a previous study (He et al., 2017). These results indicated that bacterial fitness cost could not cause plasmid loss.

The genetic relationship of the *mcr*-*1-*positive *E. coli* isolates was analyzed by MLST, which revealed that the most common ST was ST10, followed by ST48, ST20, ST3944, ST772, ST5229, ST617, ST410, ST93, ST898, and ST1421, and then by single ST isolates. More importantly, three predominant STs (ST10, ST48, and ST617) identified in the current study are different by one or two alleles and they correspond to clonal complex CC10. This result supported the previous finding that the most prevalent ST was ST10 in an investigation of *mcr*-positive *E. coli* isolated from diseased food animals in Europe (Garch et al., 2016). As we all know, ST10 is described as one of the predominant *E. coli* lineages, which is widespread among humans and animals, especially in livestock animals (Manges et al., 2015). By phylogenetic group classification, a total of 46 (82.14%) *mcr-1*-positive *E. coli* belong to groups A and B1 in this study, indicating that most of the swine-origin *mcr-1*-positive *E. coli* were non-pathogenic or commensal strains, consistent with a previous study (Khanawapee et al., 2020).

## Conclusion

The findings of this study demonstrated the high prevalence of *mcr-1* in swine farms in northeastern China. *mcr-1*-positive *E. coli* showed extensive antimicrobial resistance profiles with the presence of additional resistance genes, increased expression of efflux pump-associated genes, and increased biofilm formation ability. The high diversity of clones and the results of the conjugation experiment underlined the evidence for the horizontal transfer of *mcr-1*. The *mcr-1-*harboring plasmids could reduce the fitness of bacterial hosts but remained stable in the recipient strain. Due to the last-line role of colistin in the treatment option against infection caused by MDR GNB, and livestock production has been described as one of the greatest reservoirs of *mcr-1*, careful monitoring of the spread of *mcr-1* gene in food animals is urgently needed, particularly in swine.

## Data Availability Statement

The original contributions presented in the study are included in the article/supplementary material, further inquiries can be directed to the corresponding author/s.

## Author Contributions

XZ, YY, and PC conceived and designed the experiments. PC, SC, HL, XL, JS, FL, and MI collected the samples and performed the experiments. PC and MI analyzed the data and wrote the manuscript. All authors have read and agreed to the published version of the manuscript.

## Conflict of Interest

The authors declare that the research was conducted in the absence of any commercial or financial relationships that could be construed as a potential conflict of interest.

## Footnotes

1 http://www.ncbi.nlm.nih.gov/blast

2 https://itol.embl.de/
